# Molecular Markers of Mechanosensation in Glycinergic Neurons in the Avian Lumbosacral Spinal Cord

**DOI:** 10.1523/ENEURO.0100-22.2022

**Published:** 2022-09-13

**Authors:** Kathryn E. Stanchak, Kimberly E. Miller, Eric W. Lumsden, Devany Shikiar, Calvin Davis, Bingni W. Brunton, David J. Perkel

**Affiliations:** 1 Department of Biology, University of Washington, Seattle, WA; 2 Department of Psychology, University of Washington, Seattle, WA; 3 Department of Biology, University of Washington, Seattle, WA 98195; 4 Department of Otolaryngology, University of Washington, Seattle, WA

## Abstract

Birds are exceptionally adept at controlling their body position. For example, they can coordinate rapid movements of their body while stabilizing their head. Intriguingly, this ability may rely in part on a mechanosensory organ in the avian lower spinal cord called the lumbosacral organ (LSO). However, molecular mechanotransduction mechanisms have not been identified in the avian spinal cord. Here, we report the presence of glycinergic neurons in the LSO that exhibit immunoreactivity for myosin7a and espin, molecules essential for function and maintenance of hair cells in the inner ear. Specifically, we find glycinergic cell bodies near the central canal and processes that extend laterally to the accessory lobes and spinal ligaments. These LSO neurons are reminiscent of glycinergic neurons in a recently-described lateral spinal proprioceptive organ in zebrafish that detects spinal bending. The avian LSO, however, is located inside a series of fused vertebrae called the synsacrum, which constrains spinal bending. We suggest the LSO may be a modification and elaboration of a preexisting mechanosensory spinal network in vertebrates. A mechanistic understanding of its function may be an important clue to understanding the evolution and development of avian locomotion.

## Visual Abstract

**Figure:**
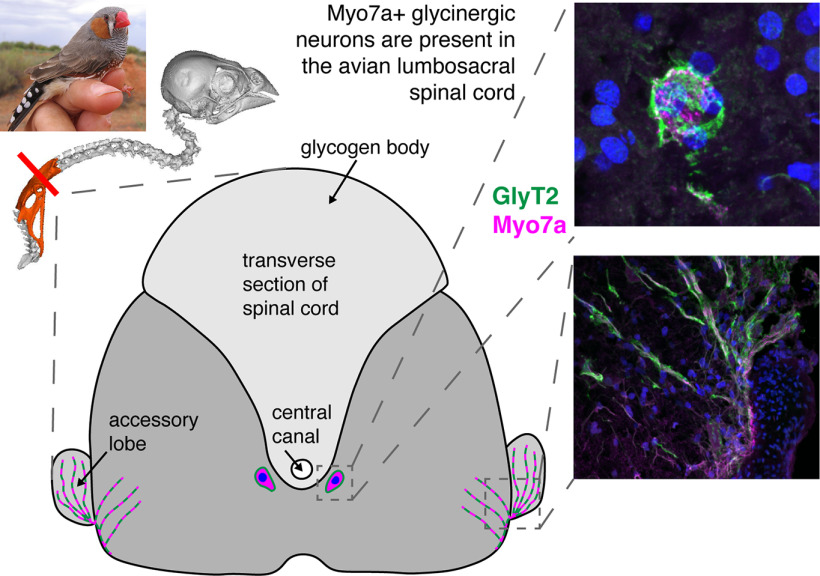

## Significance Statement

The spinal cord can help control posture by sensing bending of the spinal column. In birds, the lower spinal column is fused and cannot bend. Notably, this region of the avian spinal cord has several specialized features compared with other vertebrates. We report neurons in the lower bird spinal cord that express proteins known to be involved in sensing mechanical signals. These neurons share characteristics of other spinal cord mechanosensory cells. This suggests that spinal cords across vertebrates are capable of mechanosensation and that spinal mechanosensory networks have evolved to allow unique functions, such as in the constrained lower spinal cord of birds. These data provide an avenue for molecular manipulations of these cells, permitting experimental tests of their role in controlling posture.

## Introduction

Animals must be able to sense the position of their body to execute targeted movements, especially in response to environmental perturbations. Birds are often noted for their exceptional body-positioning abilities, including keeping their head still while their body twists and turns in response to wind or a swaying perch. As in other vertebrates, the vestibular organs of the inner ear and distributed proprioceptors affiliated with the musculoskeletal system provide sensory inputs to the avian body positioning control system. Interestingly, birds are also hypothesized to have a mechanosensory organ within their highly-modified lumbosacral spinal cord, known as the avian lumbosacral organ (LSO), which may provide additional mechanosensory information (Necker, 2006). The LSO is found in all extant birds and may vary in morphology depending on a species’ locomotor habits (Stanchak et al., 2020). However, the mechanotransduction mechanisms of the LSO remain unresolved.

It is becoming increasingly clear that vertebrates have spinal neurons that can provide sensory information on body positioning by detecting spinal bending. For instance, edge cells detect stretch at the lateral margin of the lamprey spinal cord (Grillner et al., 1984). Cerebrospinal fluid-contacting neurons (CSF-cNs) within the ependymal cells surrounding the central canal—found across vertebrates—sense alterations to CSF flow in the central canal because of spinal bending (Böhm et al., 2016; Orts-Del’Immagine et al., 2020). Recently, a lateral glycinergic proprioceptive organ has been described in zebrafish that detects stretching of neural tissue near intervertebral discs as the spine bends (Picton et al., 2021).

**Figure 1.**
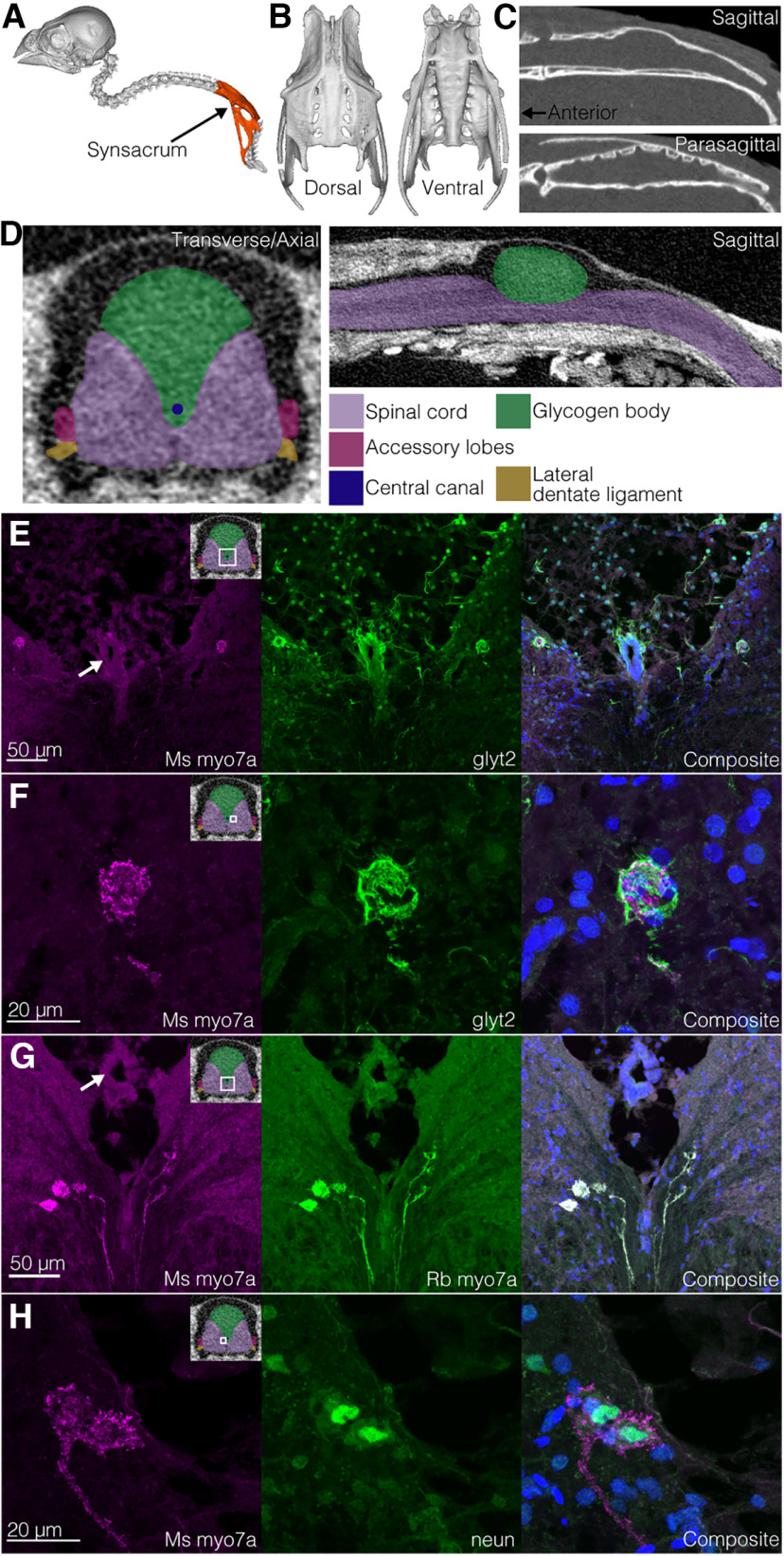
Glycinergic neuronal cell bodies near the central canal of the avian lumbosacral organ (LSO) display myosin7a immunoreactivity. ***A***, The synsacrum (fused to the pelvic bones) is highlighted within a reconstructed CT scan of the zebra finch axial skeleton (Yale Peabody Museum specimen #125076). ***B***, Dorsal and ventral views of the synsacrum, reconstructed from the CT scan. ***C***, A sagittal section through the vertebral canal of the synsacrum CT scan shows the dorsal expansion of the vertebral canal that holds the glycogen body. A parasagittal section shows transverse canal-like structures on the internal dorsal side of the vertebral canal that roughly align with the accessory lobes (see Stanchak et al., 2020). ***D***, A diagram of the neural tissue of the LSO based on dissection and histology observations and a contrast-enhanced CT scan (data from Stanchak et al., 2020). ***E***, Cells near the central canal (middle) that are immunoreactive for both glyt2 and myo7a. ***F***, Higher-resolution image of the right cell from ***E***. ***G***, Labeling with two different myo7a antibodies (mouse and rabbit). ***H***, neun/fox3 labeling within the myo7a+ cells. White arrow in the first panel of ***E*** and ***G*** points to the ependymal layer surrounding the central canal. Upper right diagram in the first panel of ***E–H*** indicates approximately where in the transverse section the image was taken. In composite images of ***E–H***, blue is DAPI.

**Figure 2.**
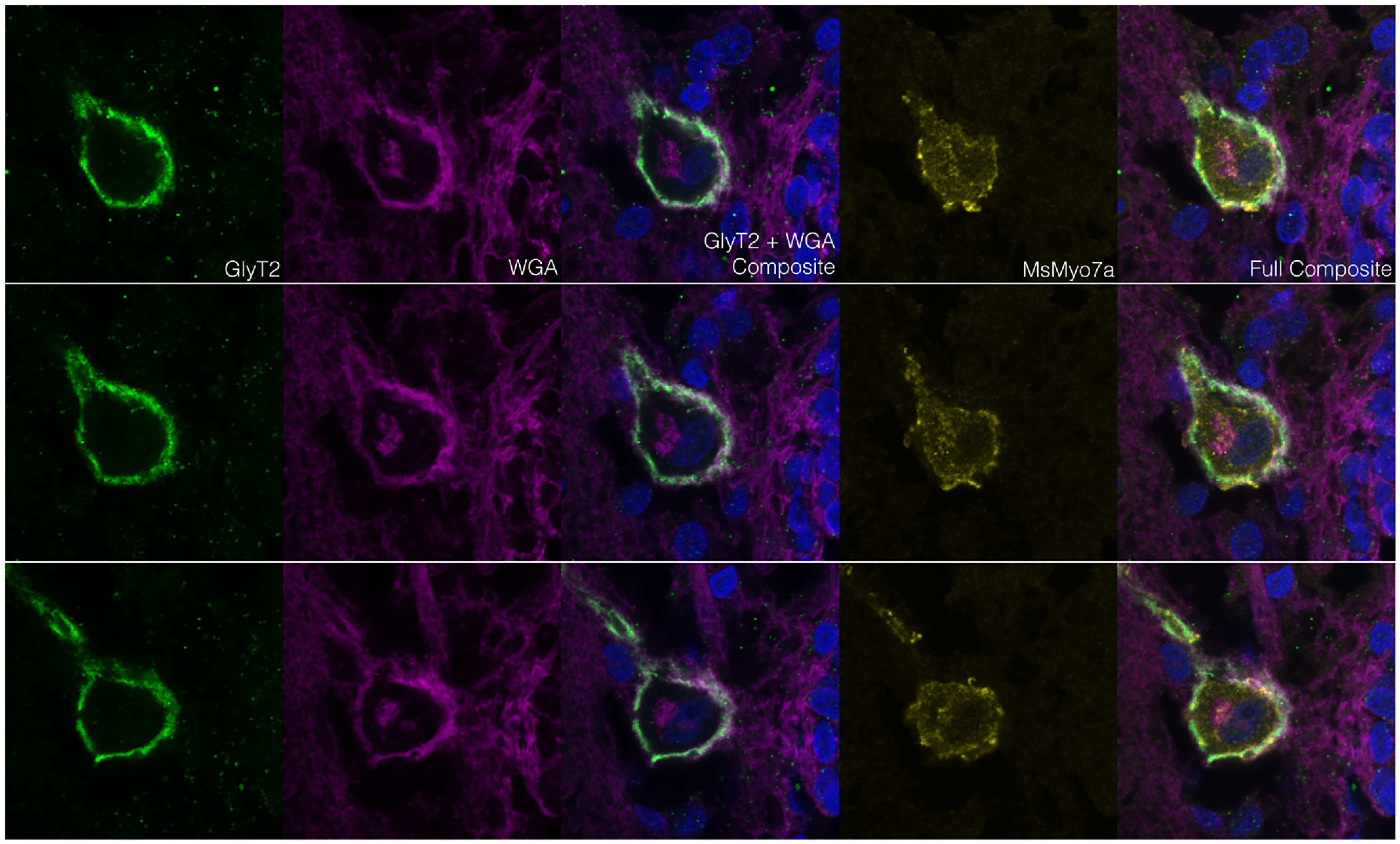
Glycine transporter 2 is located in the cell membrane of the neuronal cell bodies that display myosin7a immunoreactivity. Each horizontal row of images is a separate optical section along the *z*-axis of one cell. WGA is wheat germ agglutinin, a lectin that labels plasma membranes. In composite images, blue is DAPI. Nuclei of the ependymal cells of the central canal can be seen in the lower right of the composite images.

While these examples of spinal mechanosensory neurons all detect bending of the spine, the avian LSO lies within the synsacrum, a rigid series of fused vertebrae that prevent the spinal cord from bending (Fig. 1*A–D*). Indeed, the LSO has a striking set of specializations that make it anatomically distinct from the rest of the spinal cord. First, a glycogen body sits within a dorsal split of the spinal cord. The central canal passes through the base of the glycogen body such that the ependymal cells surrounding the central canal are fully encapsulated by glycogen body tissue (Fig. 1*D*,*E*). Second, the spinal cord has segmentally-repeating, laterally-projecting accessory lobes, which are protrusions of neural tissue that roughly align with transverse, canal-like recesses in the surrounding bone (Fig. 1*D*). Modified spinal membranes route cerebrospinal fluid through these recesses (Necker, 2006). Third, the entire neural structure is suspended within the synsacrum by a modified dentate ligament. At the ventrolateral corners of the spinal cord, the accessory lobes and the lateral dentate ligament adjoin the white matter (Figs. 1*D*, 2*A–C*). Based on these anatomic specializations, the LSO is hypothesized to work by transducing cerebrospinal fluid flow through mechanosensory neurons in the accessory lobes (Necker, 1999) or by acting as a preloaded vibratory structure (Kamska et al., 2020).

While some cellular-level neuroanatomy of the LSO has been described, little is known about the molecular features of putative mechanosensory cells. In 1905, Imhof (1905) illustrated cells and processes particular to the avian LSO, including around the central canal. Accessory lobe neurons project contralaterally, then both rostrally and caudally (Eide, 1996; Eide and Glover, 1996; Necker, 2006). Neurons within the accessory lobes produce a number of neurotransmitters, including glycine, glutamate, and GABA, as well as the enzyme choline acetyltransferase (Milinski and Necker, 2001). Processes from the neurons within the accessory lobes project into local lacunae, which are hypothesized to be part of a mechanosensory mechanism (Rosenberg and Necker, 2000, 2002; Necker, 2002). To date, no molecular mechanotransduction molecule or mechanism has been identified in the LSO.

Here, we describe neurons within the avian LSO that have molecular characteristics of known mechanosensory cells and are well positioned to transduce mechanical signals. These results provide critical evidence supporting the hypothesis that the LSO has a role in proprioception and balance in birds.

## Materials and Methods

### Tissue preparation

The spinal column was harvested from zebra finch (*Taeniopygia guttata*) individuals of either sex used for other experiments. Most were postfixed in 4% paraformaldehyde in phosphate buffer (PB) for 24 h. Results from seven individuals are reported here. Two spinal columns were alternatively harvested from transcardially-perfused individuals. In most samples, the LSO region of the spinal cord was dissected from the surrounding synsacrum bone and placed in 20% sucrose in PB for a minimum of overnight. The region of the LSO surrounding the glycogen body was then sectioned transversely at 20 μm on a cryostat in four alternating series with negative controls. For two samples (labeled with mouse-antimyo7a + rabbit-antiglyt2 and mouse-antimyo7a + rabbit-antimyo7a), the synsacrum bone was first decalcified using 12% EDTA neutralized with NaOH before the entire spinal column was sectioned. We saw no difference in reported results between decalcified and undecalcified specimens, but we note that anti-myo7a labeling seemed sensitive to extended time prefreezing in other samples tested but not reported here.

### Immunohistochemistry

Sections were briefly dried then washed 3× in 0.1% Triton X-100 in PBS (PBST) before they were blocked with 5% neutral goat serum (Abcam or Vector Labs). Sections were then incubated for double-labeling experiments with primary antibody (Ab) diluted in PBS, washed 3× in PBST, incubated with secondary Ab, then washed in PBST, PBS, and dH_2_O and incubated for 10 min in 1:1000 aqueous DAPI (Biotium) before mounting with Fluoromount G (SouthernBiotech). Two timing protocols were used: a short protocol with a 1-h block, overnight incubation with primary Ab, and 2 h of incubation with secondary Ab; and a long protocol with overnight block, two-night incubations with primary Ab, and overnight incubation with secondary Ab. For some Abs, we altered dilutions across experiments (noted below), but we saw no difference in reported results.

Primary Abs used were rabbit-antiglyt2 (Alomone AGT-012; 1:400 *n* = 5 or 1:200 *n* = 1; 13/14 immunogen amino acids identical to *T. guttata* glyt2 according to BLAST-P search), mouse-antimyo7a (Developmental Studies Hybridoma Bank 138–1, 1:100 *n* = 5 or 1:200 *n* = 4; avian cross-reactivity listed by manufacturer), rabbit-antimyo7a (Proteus 25–6790, 1:400 *n* = 3 1:500 *n* = 2; avian cross-reactivity listed by manufacturer), rabbit-antiespin (Invitrogen PA5-55941, 1:200 *n* = 5; labeled interior side of ependymal cells in *T. guttata* spinal cord, where espin+ apical tips of CSF-cNs are located; 54/64 immunogen amino acids identical to *T. guttata* espin according to BLAST-P search), rabbit-antineun/fox3 (Invitrogen 702022; 1:200 *n* = 3 or 1:500 *n* = 2; labeled cells in spinal cord as expected). The double-labeling experiments were rabbit-antiglyt2 and mouse-antimyo7a (*n* = 4); rabbit-antimyo7a and mouse-antimyo7a (*n* = 5); rabbit-antiespin and mouse-antimyo7a (*n* = 5); and rabbit-antineun and mouse-antimyo7a (*n* = 5). Secondary Abs used were goat anti-mouse or goat anti-rabbit AlexaFluor 488, 568, and 647 (Invitrogen; 1:300). For two of the individuals double-labeled rabbit-antiglyt2 and mouse-antimyo7a, we applied a wheat germ agglutinin (WGA) membrane label conjugated to AlexaFluor 488 at 1:200 with the secondary Ab solution.

**Figure 3.**
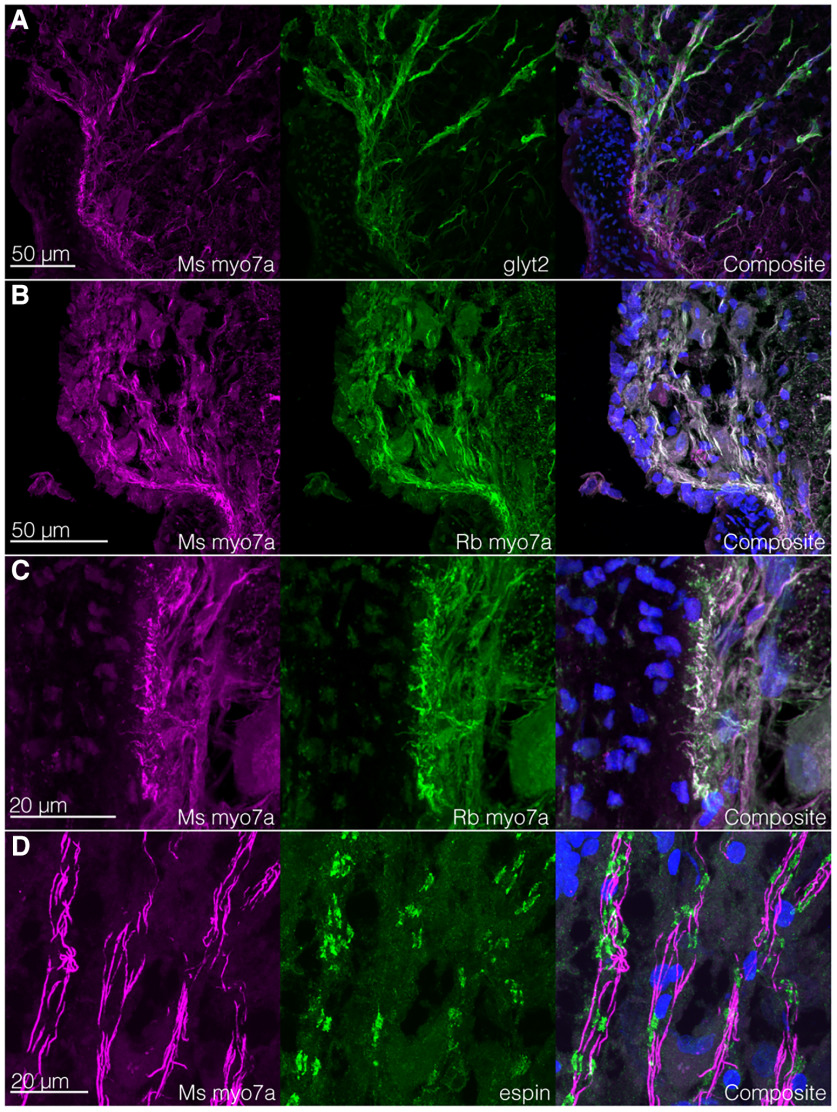
Lateral glycinergic processes are immunoreactive for proteins also present in inner ear hair cell stereocilia. ***A***, Glyt2+ ventrolateral processes are immunoreactive for myo7a. Margin of spinal cord with dentate ligament is at lower left; upper left is accessory lobe. ***B***, ***C***, The accessory lobe processes (***B***) and terminations at the dentate ligament (***C***) double-label with two different antibodies for myo7a (mouse and rabbit). ***D***, Myo7a-immunoreactive processes in the accessory lobe also react with anti-espin. ***A–D***, In composite images, blue is DAPI. Upper right diagram in the first panel of each subfigure indicates approximately where in the transverse section the image was taken.

Slides were viewed and imaged on Nikon Eclipse epifluorescence, Olympus FV1000, and Nikon A1R confocal microscopes. All images presented in the text are confocal (Figs. 1, 3, Olympus FV1000; Fig. 2, Nikon A1R).

## Results

Immunohistochemical assays of the zebra finch LSO demonstrated medial neuronal cell bodies and ventrolateral processes that expressed the sodium-dependent and chloride-dependent glycine transporter 2 (glyt2). The glycinergic medial cell bodies tended to be found near the central canal but not within the ependymal cell layer or the glycogen body that surrounds the central canal (Fig. 1*D*,*E*). Further immunohistochemical assays demonstrated that these medial glycinergic cells were also immunoreactive for myosin 7a (myo7a; Fig. 1*E–G*), an atypical myosin essential for proper inner ear hair cell function (Li et al., 2020) and neun/fox3, a neuronal cell body marker (Fig. 1*H*). Glyt2 expression in the myo7a+ cells of interest colabeled with the membrane-binding wheat-germ agglutinin, indicating that glyt2 is in the membrane of and not in presynaptic terminals adjacent to the myo7a+ cell (Fig. 2).

The glycinergic (glyt2+) ventrolateral processes traversed the gray matter to the white matter. Some processes extended into the accessory lobes, and others terminated at the margin between the dentate ligament and the spinal cord (Fig. 2*A*). These processes were also immunoreactive for myo7a (Fig. 2*B*,*C*). The processes within the accessory lobes were immunoreactive for both myo7a and espin antibodies, which in transverse sections colocalized in an alternating pattern (Fig. 2*D*). Espin is an actin-bundling protein, especially in sensory cells (Sekerková et al., 2011). Two different antibodies (from different manufacturers) for myo7a colabeled cellular structures (Figs. 1*F*, 3*B*,*C*). It was unclear from our sections whether these lateral processes extend from the medial cell bodies.

## Discussion

The putative mechanosensory neurons in the avian LSO described here comprise a lateral group of neurons that express glyt2, as in the lateral spinal proprioceptive organ of zebrafish (Picton et al., 2021). We suggest these glyt2+ neurons in the avian LSO may be a modified version of the zebrafish lateral glycinergic proprioceptive organ. Notably, there is evidence that these lateral mechanosensory zebrafish cells initially develop from progenitors near the central canal (Picton et al., 2021), which may be a developmental connection between the medial avian cells described here and the zebrafish cells.

There is no clear connection between CSF-cNs and the glyt2+ cells reported here. CSF-cNs can be found ectopically (outside of the ependymal layer surrounding the central canal (Tonelli Gombalová et al., 2020; Jurčić et al., 2021). However, a recent transcriptome analysis of spinal dynorphin-lineage cells found a cluster with characteristics of CSF-cNs but comparatively low expression of glyt2 transcript (SLC6A5; Serafin et al., 2021, their Fig. 2). CSF-cNs are known to be GABAergic (Stoeckel et al., 2003; Wyart et al., 2009; Djenoune et al., 2014); a direct comparison of GABA and glycine in CSF-cNs and these avian glycinergic cells would be helpful for distinguishing whether or not these cells share a lineage. Pkd2l1, polycystic kidney disease 2-like 1 protein, is a known marker of CSF-cNs and is a required component of the mechanosensing mechanism in zebrafish (Sternberg et al., 2018). An assessment of pkd2l1 expression in the avian glycinergic neurons reported here would also be clarifying, although to our knowledge, as of this writing there are no commercially-available antibodies for pkd2l1 for avian tissue. The characteristics we have reported here could guide future explicit lineage tracing or single-cell characterization, facilitating a more thorough comparison with CSF-cNs.

Our observations that glycinergic neurons in the LSO have immunoreactivity for myo7a and espin are critical evidence of their mechanosensory potential. Both myo7a and espin are found in inner ear hair cell stereocilia, and myo7a is directly implicated in mechanotransduction in hair cells (Sekerková et al., 2011; Li et al., 2020). In addition, espin is also found in the cilia of CSF-cNs (as is the atypical myosin 3b; Desban et al., 2019). The CSF-cN cilium is essential for skilled locomotor activity (Gerstmann et al., 2022). The presence of myo7a and espin in these LSO glycingeric neurons does not, however, explain how mechanical force may be transduced into electrical signals, i.e., the key force-transducing ion channel involved in mechanosensation is still unknown. In hair cells, mechanically-gated channels convert mechanical forces to electrical signals (for review, see Maoiléidigh and Ricci, 2019). The transmembrane channel-like proteins 1 and 2 (TMC 1/2) have been identified as the mechanosensitive components of the mechanoelectrical transduction apparatus in hair cells (Kurima et al., 2015; Jia et al., 2020). Piezo1 and piezo2 are mechanically-activated components of transmembrane channels (Coste et al., 2010, 2012) involved in homeostatic and somatosensory functions, respectively (for review, see Poole, 2021; Szczot et al., 2021). Pkd2l1, the marker of CSF-cNs mentioned above, is part of the polycystic family of transient receptor potential (TRP) channels, many of which are implicated in mechanotransduction (for review, see Christensen and Corey, 2007; Delmas et al., 2011). Pkd2l1 is required for CSF-cN function in zebrafish (Böhm et al., 2016; Sternberg et al., 2018), but interestingly does not appear to be required for CSF-cN function in mice (Gerstmann et al., 2022). Both protein and transcript assays for the above candidate channels (TMC 1/2, piezo2, pkd2l1) as well as putative mechanosensitive current properties of LSO glycinergic neurons could help identify the molecular structure of a mechanotransduction apparatus.

The fused vertebrae of the bird synsacrum prevent the lower spine from bending; therefore, the CSF-cNs and lateral proprioceptive spinal organ cannot function as has been described in other vertebrates. Rather, the neuronal processes in the accessory lobes may sense cerebrospinal fluid flow (as hypothesized and summarized by Necker, 2006), while the processes at the junction between the spinal cord and the dentate ligament may detect differential vibration among tissue types (according to the vibration/suspension hypothesis by Kamska et al., 2020).

Our finding of the presence of myo7a-immunoreactivity and espin-immunoreactivity in LSO neurons is a starting point that critically enables testing of cellular, organ, and organismal-level mechanotransduction mechanistic hypotheses. Myo7a in particular may be a promising target for molecular manipulations. The ability of birds to impeccably control their body position may have helped them diversify into a clade with multifunctional locomotor specializations, including the capacity for terrestrial bipedalism as well as flight. Thus, continued research on the avian LSO may reveal foundational physiological and developmental mechanisms that enabled the evolution and diversification of avian locomotion.

## Synthesis

Reviewing Editor: David Schoppik, New York University - Langone Medical Center Decisions are customarily a result of the Reviewing Editor and the peer reviewers coming together and discussing their recommendations until a consensus is reached. When revisions are invited, a fact-based synthesis statement explaining their decision and outlining what is needed to prepare a revision will be listed below. The following reviewer(s) agreed to reveal their identity: Eve Schneider, Abdeljabbar El Manira.

In brief, the reviewers and I think the manuscript could more convincingly make the case for colocalization. Mechanotransducer cells are often so closely apposed to other cells (satellite cells, lamellar cells, etc...) that without sufficient image resolution (particularly in the z axis- i.e., using confocal rather than epifluorescence) it is difficult to tell if this signal is from colocalized structures or just two different cells very close together. We’re all hoping you’ve got the samples available for a quick imaging session. Everything else can be dealt with in the text.

1) The data shown to demonstrate colocalization of myo7a and glyt2 does not seem convincing. Specifically, the close-up in figure F does not look very colocalized. As many sensory neurons (for instance DRG neurons) are often surrounded by satellite glial cells, it is not clear whether this is a glyt2 positive process in close contact with a myo7a neuron, or whether they are truly in the same neuron. Figure 2A is somewhat more convincing, so this may just be a case of choosing another representative example, or slightly redoing these imaging experiments with modified methods. In the methods the section thickness is not mentioned, nor whether this particular image was obtained via confocal or epifluorescence. This should be corrected. In addition, a higher magnification/resolution image of a thin section (if using epifluoresence) or a confocal section (ideally deconvolved), would go a long way toward convincing the reader that these proteins are really in the same neuron. Showing a few more example images, or reporting quantification of colocalization in the text would further demonstrate this point. Since it is likely that Myo7a and glyt2 are not expressed in the same subcellular region using an alternate marker that is expressed in the plasma membrane of these neurons could also be a useful approach.

2) While the authors state that these glycinergic neurons are not CSF-cNs’s because those express GABA across vertebrates, they also present no negative evidence of these neurons expressing any GABA-synthetic components, so it is not clear that they can rule out that these neurons don’t also express GABA in addition to glycine. Without showing these neurons are not capable of synthesizing or releasing GABA it does not seem accurate to state that these are not CSF-cNs’s. This could be addressed by changing the text or doing an additional histology experiment.

3) The molecular markers discussed (Myo7a, glyt2, espin) have been linked to mechanosensory neurons in other species, though these observations are either 1) in species that have spines that bend near the lumbosacral organ or 2) are in hair cells. The authors state that Myo7a is “directly linked to mechanotransduction in hair cells,” with appropriate references. However, for a neuro to be mechanosensitive it must have at least one force transducing molecule. I don’t think the author is arguing that myo7a is the force transducer in hair cells, but it would be great to see some discussion on what might be the likely force transducer. For instance, some of these CSF-contacting neurons (CSF-cNs) express PKDL21 (Böhm et al., Nat Comm, 2016), but any other evidence from the literature or the author’s observation of what channels that might be involved in mechanotransduction of CSF-cNs’s, such as Piezo or TMC1/2, etc... would be really interesting! Whilst the authors identify clear markers associated with mechanosensation (e.g. myo7a, espin) it remains unclear what the actual mechanosensitive proteins in these neurons might be. Alternatively, the study would benefit from more data (e.g. Piezo2) or discussion on the potential mechanosensitive proteins expressed by these neurons.

4) The authors correctly point out the fused synsacrum, and thus ruling out sensing bending of the spinal cord as the mechanism of force sensing in these neurons, instead positing that these neurons respond to CSF flow. They make no explicit conjectures about how CSF-cNs’s sense CSF flow, which makes sense because their data does not explicitly indicate anything about this mechanism, other than citing ref’s 10 and 7. However, they seem to be in a strong position to make such statements, perhaps with further analysis of their existing images- for instance, do the glyt2/myo7a neurons extend microvilli into they could be bent by fluid flow? Are they tethered to other structures, such as membranes? Do they possess any other specialized endings, such as corpuscles? It would be useful to see more attention paid to anatomical specializations of the CSF-cNs morphology that could generate predictions about how these neurons might be sensing the flow of ACSF. The authors mention in the methods that they did not see a difference between intact and decalcified samples, for a morphological analysis of these neurons one might expect more difference, so if further analysis of morphology is included this should be considered.

5) Glycinergic neurons, with mechanosensitive features, have been described before in the chick and pigeon spinal cord. For example there have been previous descriptions of their commissural, ascending axon projections (Eide 1996) and their intrinsic mechanosensitivity to vibratory stimuli (Necker 2002). Are these neurons the same or a different population as described in this study? The manuscript would be improved by clarifying what has been previously shown for different neuronal subpopulations within the LSO that are proposed to be mechanosensitive, including key references that are currently missing. Similarly, Some discussion on whether LSO neurons are similar to cerebrospinal fluid contacting neurons described in lamprey and larval zebrafish or to the intraspinal proprioceptors described in adult zebrafish would be beneficial.

Minor comments:

1)Page 2, Results, Para 2. There seems to be some referencing issue here

"[?, e.g.,]]sekerkova2011roles.” There is a typo in results section in Serkova reference (maybe /citep can’t deal with the “e.g.?”).

2) What age were the animals that used? Please specify and also discuss if there could be developmental changes, for example in the position of these neurons.
